# Cross-linked enzyme aggregates (CLEAs) of selected lipases: a procedure for the proper calculation of their recovered activity

**DOI:** 10.1186/2191-0855-3-25

**Published:** 2013-05-12

**Authors:** María del Pilar Guauque Torres, María Laura Foresti, María Luján Ferreira

**Affiliations:** 1 Biocatalysis Group, Planta Piloto de Ingeniería Química (PLAPIQUI), Universidad Nacional del Sur – CONICET, Camino La Carrindanga Km 7 CC 717 (8000), Bahía Blanca, Argentina; 2 Polymer and Composite Materials Group, Instituto de Tecnología y Ciencias de la Ingeniería (INTECIN), Facultad de Ingeniería, Universidad de Buenos Aires – CONICET, Las Heras 2214 (1127AAR), Buenos Aires, Argentina

**Keywords:** CLEAs, Lipase, Precipitable protein, Specific activity, Recovered activity

## Abstract

In the last few years, synthesis of carrier-free immobilized biocatalysts by cross-linking of enzyme aggregates has appeared as a promising technique. Cross-linked enzyme aggregates (CLEAs) present several interesting advantages over carrier-bound immobilized enzymes, such as highly concentrated enzymatic activity, high stability of the produced superstructure, important production costs savings by the absence of a support, and the fact that no previous purification of the enzyme is needed. However, the published literature evidences that a) much specific non-systematic exploratory work is being done and, b) recovered activity calculations in CLEAs still need to be optimized. In this context, this contribution presents results of an optimized procedure for the calculation of the activity retained by CLEAs, based on the comparison of their specific activity relative to their free enzyme counterparts. The protocol implies determination of precipitable protein content in commercial enzyme preparations through precipitation with ammonium sulphate and a protein co-feeder. The identification of linear ranges of activity versus concentration/amount of protein in the test reaction is also required for proper specific activity determinations. By use of mass balances that involve the protein initially added to the synthesis medium, and the protein remaining in the supernatant and washing solutions (these last derived from activity measurements), the precipitable protein present in CLEAs is obtained, and their specific activity can be calculated. In the current contribution the described protocol was applied to CLEAs of *Thermomyces lanuginosa* lipase, which showed a recovered specific activity of 11.1% relative to native lipase. The approach described is simple and can easily be extended to other CLEAs and also to carrier-bound immobilized enzymes for accurate determination of their retained activity.

## Introduction

Biocatalysts (enzymes and whole cells) offer high activity and chemo-regio-stereo-selectivity under mild reaction conditions which would be impossible for proper performance of traditional chemical catalysts. However, usually, the wide use of soluble enzymes in industrial processes is restricted due to their high cost, low operational stability, and their difficult recovery and reuse. Immobilization of enzymes increases their stability to the denaturing effect of temperature and -in some cases- of organic solvents, and allows easier recovery and reuse. Unfortunately, it has some negative aspects such as increased costs (the cost of the support itself and the cost of the technology involved in the immobilization process), the possibility of internal diffusion limitations (if the enzyme is immobilized inside the pores of the support material), and changes in enzyme activity caused by distortion of its tridimensional structure after immobilization. Besides, when carrier-bound enzymes are used a significant dilution of specific activity is observed since between 90-99% of the percentage in mass of the immobilized enzyme is carrier (Cao et al. 2003).

In the early 1960´s, “carrier-free enzyme immobilization” was attempted. The first step in this type of insolubilization was the development of the so-called insoluble amorphous cross-linked enzymes (CLEs) produced by reaction of the NH_2_ groups of the enzyme. CLEs were soon followed by the development of cross-linked enzyme crystals (CLECs), which implied crystallization of the enzymes and their cross-linking with glutaraldehyde (Sheldon 2007a). The main disadvantage of CLECs was the need to purify and crystallize the enzyme. In the year 2001, and as a possible solution for CLECs problems; the so-called cross-linked enzymes aggregates (CLEAs) were developed (Cao et al. 2001). CLEAs result from the cross-linking of physical aggregates of enzymes molecules. The procedure of the synthesis of CLEAs starts with the formation and precipitation of enzymatic protein aggregates (without perturbation of their tertiary structure), caused by the addition of organic solvents, non-ionic polymers or salts to an aqueous solutions of proteins. The formation of the physical aggregates occurs by a change in the hydration state of enzyme molecules and/or by altering the electrostatic constant of the solution. Later on, the physical aggregates are given a more stable superstructure using a cross-linking agent. This agent establishes covalent bonds between enzymatic protein molecules, rendering them permanently insoluble (Sheldon 2007b). The methodology is applicable to essentially any enzyme. Indeed, Sheldon et al. (2006) obtained CLEAs of penicillin acylase (the first CLEAs obtained), esterases, trypsin, oxynitrilases, nitrilases, galactosidase, catalase, lacasses and other enzymes (Sheldon et al. 2006). The advantages of CLEAs are high volumetric activity (the enzymatic activity is not diluted in the carrier), lower production costs (there is no need of a purification step and no carrier is used), and the simplicity and broad applicability of the insolubilization technique. Besides, unaltered activity of CLEAs in organic solvents has been reported, which allows high stability in multiple reactions, their recovery and reuse (Cao 2005).

As it has been pointed out in the recent article of Gardossi et al. (2010), in which guidelines for the reporting of biocatalytic reactions are given, ideally *specific activity values* (mol of substrate converted or product produced per time unit and per amount of enzyme) should be given when enzymatic activity is reported. However, to do so for CLEAs, and for comparing the obtained value with the specific activity of the ensuing native enzyme, several conditions should be met. In the first place, for both free enzyme and CLEAs enzymatic activity should be measured within linear ranges of activity-enzyme amount/concentration that guarantee constant specific activity. Besides, the amount of protein present in the commercial enzyme solution used, and in the produced CLEAs must be known. Finally, the same reaction time and experimental conditions must be used in both assays, which implies that temperature, pH, substrate concentrations, agitation, reaction volume, reactor configuration, etc., need to be the same when native enzyme and CLEAs are assayed. In the following paragraphs a method for accurate determination of the recovered enzymatic activity of CLEAs that includes all the mentioned requirements is proposed for the first time. The methodology described is exemplified with the quantification of the recovered activity of CLEAs of *Thermomyces lanuginosa* lipase (TLL) by use of triolein hydrolysis as the reaction test. The approach can be easily extended to other reactions.

## Materials and methods

### Enzymes

Commercial solutions of Lipozyme CALB from *Candida antarctica B* (CALB, 5000 U/mL) and Lipozyme TLL from *Thermomyces lanuginosa* (TLL, 5000 U/mL), were donated by Novozymes (Bagsvaerd, Denmark). Powdered commercial preparations of *Candida rugosa* lipase (CRL, 64000 g/mol, 30000 U/g), *Pseudomonas fluorescens* lipase (PFL, 33000 g/mol, 25800 U/g) and *Pseudomonas cepacia lipase* (PS,33000 g/mol, 23000 U/g) were kindly donated by Novozymes A/S. Bovine serum albumin (BSA) 30% w/v was purchased from Wiener Lab (Argentina).

### Chemicals

Triton X-100, buffer Tris–HCl 1M and triolein (65%) were purchased from Sigma Aldrich. Ethanol (99%) was obtained from Dorwill, acetone and ammonium sulphate were both from Cicarelli. Glutaraldehyde solution (25 v/v) was from Fluka and it was used as received.

### Determination of precipitable protein (PP) in commercial enzyme solutions

Saturated ammonium sulphate solution (prepared at 0°C) (variable amounts from 1:1 to 1:6 enzyme solution: salt solution v/v), was poured into appropriate vessels containing 1 mL of commercial enzymatic solution until complete precipitation took place. Precipitation was performed in an ice bath with gentle magnetic stirring during 5 h. The obtained precipitates were recovered and dried in vacuum at ambient temperature until constant weight was verified. The mass of precipitates obtained corresponds to the mass of precipitable protein (PP) present in 1 mL of the commercial solution of enzymes.

### Test reaction: Hydrolysis of triolein

The hydrolysis of triolein used as test reaction was performed following a procedure adapted from Rocha et al. (1999). The reaction mixture consisted of triolein (0.5 g), triton X-100 (2.5 g), buffer Tris–HCl 1 M dilution 0.1 in water (5 g), and distilled water (2 g). The mixture was pre-incubated at 45°C for 10 min with continuous stirring at 1000 rpm. Then, a proper amount of the target sample (commercial enzyme solution/powder, synthesized CLEAs, residual supernatant, or recovered washing solutions) was added and the reaction started. After 5 min of reaction, hydrolysis was quenched by addition of ethanol (20 mL). Enzymatic activity was calculated by determination of liberated fatty acid through titration with 0.05 N KOH and using phenolphthalein as end-point indicator. Experiments were performed twice with an average relative error of 2%. Specific activity is defined as μmol of oleic acid liberated per minute of reaction and mg of PP. The described test permitted the determination of activity of free lipases and CLEAs, as well as that of supernatants and washing solutions, which in turn allowed the calculation of the PP present in the CLEAs by mass balance.

### Determination of linear ranges of activity-protein mass

Increasing quantities of the commercial enzyme preparations were assayed in the hydrolysis of triolein under the conditions detailed in the previous section. Activity versus mass of PP graphs were constructed to determine ranges in a which linear response was guaranteed.

### Preparation of CLEAs of lipase

Commercial lipase solution (120 μL) and BSA (170 μl 30% w/v - 1:2 lipase:BSA wt/wt) were homogenized for 10 min at 500 rpm in an ice bath. Saturated ammonium sulphate solution (700 μL, 500 mg/mL) was then added and precipitation began. After 2 h, glutaraldehyde solution (200 μL, 25% v/v) was added and cross-linking was allowed to continue for the following 2 h. Then, the solution was centrifuged for 5 min at room temperature and 6000 rpm. The supernatant was decanted and the precipitate was washed with distilled water three times (0.5 mL). The obtained CLEAs were vacuum-dried at room temperature for 16 h. Residual supernatant and washing solutions were recovered and stored in the refrigerator for later quantification of PP content.

## Results

**Figure 1:**
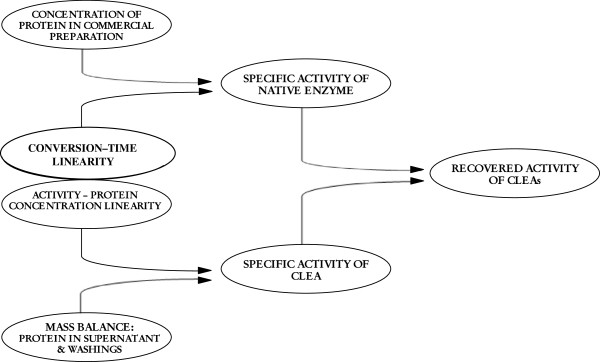
Scheme of calculation of recovered enzymatic activity in CLEAs.

Enzymatic activity of CLEAs is often reported in terms of recovered or retained activity relative to free enzyme (Sheldon et al. 2006; Lopez-Serrano et al. 2002; Matijosyte et al. 2010). However, in order to do so in a proper way, activities measured for both native enzyme and CLEAs need to be determined under *identical appropriate* reaction conditions. Besides, for a fair comparison of activity between native enzyme and CLEAs, the enzymatic protein content of both of them should be the same. Thus, rigorous conclusions of for example, loss of activity or hyperactivation of CLEAs respect to native enzyme, can be drawn. In this context, specific activities appear as the key for guaranteeing that comparisons of activity between free enzyme and CLEAs are done based on a similar amount of protein. Rigorous determination of specific activity for both free and cross-linked enzymes requires that lipase activity determinations are done in linear regions of conversion-time and activity-protein mass/concentration data. As it will be explained in detail later on, mass balances that explicitly consider the mass of protein lost in supernatant and CLEAs washings need to be considered in order to properly quantify the protein present in CLEAs. Figure 1 schematizes the protocol we hereby propose for the calculation of the retained activity of CLEAs based on the comparison of specific activities of CLEAs and the specific activity of free enzyme. Data needed to determine them is summarized (concentration of protein in commercial enzyme preparation, activity measurements in intervals where linear conversion-time and activity-mass/concentration of protein responses are verified, protein lost in supernatant and CLEAs washing solutions), and they will be the focus of the following sections.

### Determination of precipitable protein (PP) in commercial enzyme solutions

Calculation of the specific activity of free enzymes may seem a trivial point, since once activity is measured under proper conditions (see Discussion on this issue later on), the value obtained has just to be divided by the amount of protein used. The protein content of enzyme preparation should be directly obtained from the information given by the supplier of the commercial preparation in terms of protein content per mL of solution or per mg of solid preparation. However, the concentration of protein present in commercial enzyme preparations is seldom provided by suppliers. Instead, suppliers often provide information in terms of Units/g or Units/mL. A Unit (U) is generally defined as the amount of a certain enzyme that catalyzes the conversion/production of 1 mmol or μmol of substrate/product per unit of time *in a specific reaction and under specific reaction conditions* (temperature, pH, substrate concentration, etc.). Table 1 exemplifies the variety of reaction types used by suppliers for reporting the activity of well-known commercial lipases.

**Table 1:** Selected examples of the different reactions used by suppliers to test lipase activity

**Commercial lipase preparation**	**Supplier**	**Physical state**	**Lipase activity**	**Test reaction**	**Definition of Unit**	**Reference**
CRL (Lipase AY 30)	Amano	powder	> 30000 U/g	Not specified hydrolysis	Amount of enzyme that releases 1 μmol of fatty acid per minute at pH 7, not specified temperature	Technical sheet
CRL (VII )	Sigma Chemicals Co.	powder	900000 U/g	Hydrolysis of olive oil	Amount of powder which produces 1 μmol of oleic acid per hour at pH=7.2 and 37°C.	Arroyo (1995)
Lipozyme RM IM (RML)	Novozymes	immobilized	> 150 U/g	Interesterification of soybean oil, no co-solvents	0.01 w/w% converted tristearin/min-g enzyme.	Technical sheet
Novozym 435 (CALB)	Novozymes	immobilized	~ 10000 U/g	Synthesis of propyl laureate	n.a.	Technical sheet

Esterification, hydrolysis and interesterification reactions are often used to determine Units of lipase present in commercial preparations. Unfortunately, many times no indication of the reaction used as test is even given, as it is for example the case of the widely used lipases from *Thermomyces lanuginosa* TLL (5000 U/mL), *Rhizomucor miehei* RML (5000 U/mL), and *Candida antarctica B* CalB L (5000 U/mL) from Novozymes; and *Pseudomonas fluorescens* PFL Lipase AK 20 (> 20000 U/g) and *Pseudomonas cepacia* PS (23000 U/g) from Amano. Since different suppliers of a certain enzyme, -in this case a certain lipase-, often use different reactions for testing their activity and then calculating the correspondent Units, comparison of commercial preparations just by the Units/mL or Units/mg they report is impossible. Moreover, and as it will be further discussed in the next section, Units measured in a specific reaction do not give any information on the activity of the biocatalyst in another reaction or even under different operation conditions. Only a correct specific activity determination in the reaction of choice may enable comparisons among different sources of a certain enzyme family.

Going back to the calculation of specific activity of free enzymes, Units supplied by providers do not give any information on the protein/enzyme/lipase concentration of the commercial preparation, so they are not useful for determining specific activities. Then, prior to calculation of the recovered activity of CLEAs, determination of the protein content of native enzyme (commercial preparation) is required. A number of methods to determine protein content in commercial preparations have been proposed, but their validity is discussable (see Discussion section later on). In the current contribution, protein content of commercial preparations of widely used lipases was attempted by precipitation from solution. Precipitation of proteins from solutions can be carried out by use of organic solvents or inorganic salts. In the current contribution, determination of precipitable protein content of *Candida antarctica B* lipase (CALB), *Thermomyces lanuginosa* lipase (TLL) and other well-known commercial lipases was attempted by use of i) acetone as representative of organic solvents (data not shown), and ii) ammonium sulphate as a representative of inorganic salts. In the case of ammonium sulphate, precipitation was optimized by addition of variable quantities of the precipitant agent until enzymatic activity recovered in the supernatant (measured in triolein hydrolysis) was minimal. Results of PP content of lipase solutions assayed in this contribution showed that, when using acetone as precipitant a very small amount of PP was recovered in absence of additives that promoted precipitation. For this reason, bovine serum albumin (BSA) was used as co-feeder and results in terms of recovered mass were certainly better (data not shown). The value of PP present in the commercial preparations was then obtained by subtraction of the amount of BSA used from the total mass recovered. In the case of the use of ammonium sulphate as precipitant for commercial enzyme solutions, it must be pointed out that during PP precipitation co-precipitation of the salt must be controlled. Washings of the precipitate are discarded since not cross-linked protein aggregates would re-dissolve. Then, the precipitation protocol should be optimized for each individual enzyme focused on minimizing both the enzymatic activity remaining in the supernatant and the co-precipitated salt. X-ray diffraction studies of the precipitate may help in the detection and quantification of co-precipitated ammonium sulphate. Table 2 illustrates the amount of protein present in a number of commercial lipase preparations according to values given in published literature, as well as those determined by use of ammonium sulphate in this contribution. Values obtained are in the range of published literature values.

**Table 2:** Amount of protein present in commercial preparations of common lipases

**Commercial lipase preparation**	**Supplier**	**Protein content (%)**	**Method of measurement**	**Reference**
CRL (VII )	Sigma Chemicals Co. (Missouri, USA)	**22**	n.a.	Arroyo (1995)
CRL (VII)	Sigma Chemicals Co. (St. Louis, USA).	**6**	Lowry	López et al. (2004)
CRL (VII)	Sigma Chemicals Co. (St. Louis, USA).	**9.3**	n.a.	Gitlesen et al. (1997)
CRL (AY 30)	Amano (Nagoya, Japan)	**3.3**	Lowry	López et al. (2004)
CRL (AY)	Amano (Nagoya, Japan)	**10**	n.a.	Gitlesen et al. (1997)
CRL (AY)	Amano (Nagoya, Japan)	**1.1**	Bradford	Salis et al. (2008)
PS (Lipase PS)	Amano (Nagoya, Japan)	**0,55**	Bradford	Salis et al. (2008)
PS (Chirazyme L-9, Lyo)	Roche Diagnostics (Germany)	**12**	Micro-Bradford (Standard:BSA)	Guieysse et al. (2004)
PFL (Lipase AY)	Amano (Nagoya, Japan)	**2**	Bradford	Salis et al. (2008)
RML (Chirazyme L-9, Lyo)	Roche Diagnostics (Germany)	**15**	Micro-Bradford (Standard:BSA)	Guieysse et al. (2004)
CALB Novozym 435	Novozymes A/S (Bagsvaerd Denmark)	**8**	Precipitation with acetone	Prabhavathi et al. (2009)
CALB SP525	Novo Nordisk Biondustrias, S.A. (Madrid, España)	**10**	Biuret	López et al. (2004)
Lipozyme CALB (Optimized value)	Novozymes	**30**	Precipitation with (NH_4_)_2_SO_4_	This work
Lipozyme TLL (Optimized value)	Novozymes	**20**	Precipitation with (NH_4_)_2_SO_4_	This work

### Determination of linear ranges in activity – mass of PP curves

Proper determination of the catalytic activity of a biocatalyst in terms of mmol/time, requires that linearity in terms of conversion-reaction time and activity-biocatalyst amount/concentration, are guaranteed (see scheme in Figure 1). The progress of biocatalytic reactions does not continue linearly indefinitely, but it tends to slow down with time due to the decreasing of substrate concentration, the increasing of product concentration, or the inactivation of the biocatalyst (Gardossi et al. 2010). Then, as for any other catalyst, single point measures of CLEAs activity and the activity of the correspondent native enzyme should only be performed in time periods that guarantee that linearity is maintained.

Similarly, for any reaction used to monitor CLEAs activity, a linear relationship between activity and added protein should be verified prior to any other assay. To do so, enzymatic activity versus PP amount or versus PP concentration must be experimentally determined. Determinations of these data are done using the enzyme in its native/free form. The results are later used for the determination of the appropriate mass of CLEAs, supernatant aliquots, and washings aliquots to be assayed so that linear responses are guaranteed, and thus target PP content can be inferred. It must be pointed out that the assay of those aliquots in the test reaction implies two basic assumptions: i) the enzyme present in the supernatant/washings has the same activity than free enzyme; ii) the composition of PP in CLEAs obtained is similar to the composition of PP found by precipitation by use of the ammonium sulphate protocol. The validity of the previous assumptions should be properly checked in each particular case.

**Figure 2:**
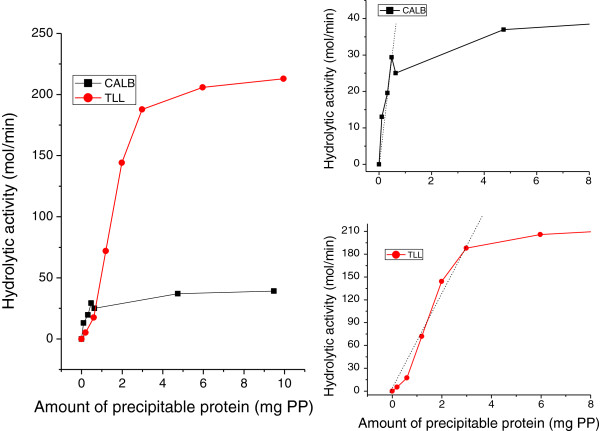
**Hydrolytic activity versus mass of precipitable protein curves found for triolein hydrolysis using commercial solutions of lipases from *Candida antarctica B *and *Thermomyces lanuginosa*.** Linear ranges are shown in the magnified image (right side).

In the current contribution the reaction used to quantify the activity of the free enzyme, CLEAs, supernatant and washing solutions from CLEAs preparation, was the hydrolysis of triolein performed in an emulsioned system. Figure 2 shows the activity profiles obtained by use of increasing amounts of commercial lipases from *Candida antarctica* B and *Thermomyces lanuginosa* in the mentioned reaction. As it is illustrated, and as also reported by Reis et al. (2010), there is a *maximum amount of enzyme reported as PP* which produces a proportional increase in activity. After that, the addition of larger quantities of enzyme does not increase the hydrolytic activity and, as a consequence, the activity per mg of PP goes down. To put it into numbers, if 50 mg of PP from TLL instead of 3 mg of PP from TLL (maximum of linear range - Figure 2) are used in triolein hydrolysis to test the activity of native enzyme, specific activity (μmol/min-mg_PP_) would decrease by a factor of 16.7. The importance of the error is enzyme and reaction dependent.

**Figure 3:**
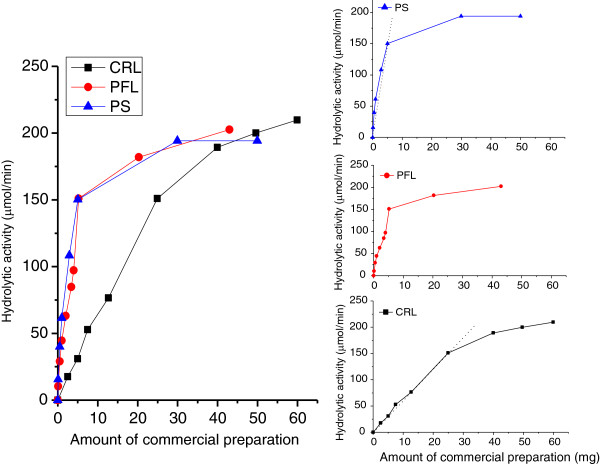
**Hydrolytic activity versus mass of commercial preparation curves found for triolein hydrolysis using powdered commercial preparations of lipases from *Pseudomonas cepacia *(PS), *Pseudomonas flourescens *(PFL) and *Candida rugosa *(CRL).** Linear ranges are shown in the magnified image (right side).

As it is illustrated in Figure 2, CALB and TLL lipases show different intervals of PP mass in which linearity is observed. Hydrolytic activity versus amount of powdered commercial preparations for other commonly used lipases, such as lipases from *Candida rugosa* (CRL)*, Pseudomonas fluorescens* (PFL) and *Pseudomonas cepacia* (PS), were determined and these data are shown in Figure 3. PFL and PS showed very similar activity responses, with linear intervals kept up to the addition of 6–7 mg of powdered biocatalyst. On the other hand, *Candida rugosa* lipase showed a lower specific activity (i.e. smaller slope in activity vs. amount of catalyst data), with a linear interval that was verified up to the addition of 25 mg of biocatalyst.

### Determination of precipitable protein (PP) in CLEAs by mass balance and calculation of their recovered activity

As introduced in the previous section, the determination of PP content in commercial lipase preparations, as well as the identification of linear ranges of activity-PP mass data (which differ among lipases as shown in Figures 2 and 3), are both of great importance for the proper calculation of the total protein present in CLEAs. Besides proteins, CLEAs contain the reacted cross-linking agent, tightly-bound water, and probably salts, organic solvents, additives and/or cofeeders remaining from the precipitation and cross-linking processes. Then, specific activity calculations need to individualize the PP present in the cross-linked aggregates. In the current contribution, quantification of total protein present in CLEAs was done by use of a mass balance which explicitly considers the fraction of protein that remained in the supernatant, as well as the fraction of protein that was washed away in each of the washing steps performed after the recovery of the CLEAs by decantation/centrifugation. Washings are aimed at removing the remaining precipitant; and they are possible at this stage since the cross-linking step performed guarantees the stability of the physical aggregates and avoids their solubilization.

To calculate the PP content of supernatant and washing solutions, activity-protein amount/concentration graphics are of great help since they i) assist in the determination of the appropriate aliquot of supernatant and washings to be assayed in order that the limit of linearity is not surpassed, and ii) allow a direct calculation of the protein content of the aliquots from the activity value registered. The selection of proper aliquots for the assay of supernatant and washings is based in the maximum volume of those that would fall in the linear range in case *all* PP added for CLEAs synthesis had remained in the supernantant or had been washed away with the washing solutions, respectively. The previous assumption guarantees that aliquots would always be measured within linear ranges. Precise measurement of the total volume of supernatant and washing solutions recovered is very important in the quantification step. Once total PP content in supernatant and washings is determined, subtraction from the total amount used for CLEAs synthesis gives the amount of precipitable protein present in CLEAs. Then their specific activity can be easily obtained. Surprisingly, that kind of procedure is not usually applied/reported in CLEAs literature, and accurate quantification of their recovered activity by further comparison with ensuing free enzyme data is severely hindered.

The procedure described for the calculation of the recovered activity of CLEAs is exemplified in Table 3 for CLEAs of TLL obtained by use of different amounts of cross-linking agent (see Materials and Methods section for further details on synthesis conditions). Intermediate calculated values needed for the determination of the retained activity of CLEAs have been summarized.

**Table 3:** Effect of variable glutaraldehyde concentrations on different quantities determined during calculation of the recovered activity of CLEAs of TLL (hydrolysis of triolein)*

**Mass of glutaraldehyde (mg)**	**Total mass of CLEAs recovered**	**Total mass of PP lost in supernatant and washings**	**Mass of CLEAs assayed (aliquot)**	**Mass of PP in aliquot of CLEAs assayed**	**Activity of CLEAs**	**Specific activity of CLEAs**	**Recovered activity of CLEAs**
	**(mg)**	**(mg)**	**(mg)**	**(mg)**	**(μmol/min)**	(μmol/min*mg)	**(%)**
10	68.7	1.35	13.1	4.3	9.5	2.2	3.5
20	83.4	0.7	12.7	3.5	11.4	3.2	5.2
40	98.2	0.07	10.3	2.5	14.4	5.8	9.2
50	95.5	0.57	11.4	2.8	19.5	7.0	11.1
60	110	6.7	12.5	1.9	9.2	4.8	7.7
80	128	4.3	13.3	2	8	3.9	6.3

* Initial mass of PP used for CLEAs´ synthesis: 23.8 mg. Specific activity of native TLL under identical conditions: 62.9 μmol/min-mgPP.

The methodology used allowed the calculation of the fraction of specific activity retained by the cross-linked aggregates (Column “Recovered activity of CLEA” in Table 3), which –although low-, evidenced a maxima for CLEAs prepared with 50 mg of glutaraldehyde. To better describe the calculations performed, details for CLEAs synthesized with 50 mg of glutaraldehyde as cross-linker will be briefly given. The volume of commercial preparation used for the synthesis of CLEAs of TLL was 120 μl, which according to Table 2 contains 23.8 mg of PP. After the centrifugation, washing and drying steps the total mass of CLEAs recovered was 95.5 ± 2.6 mg (Column “Total mass of CLEA recovered” in Table 3). The activity of CLEAs was tested by using an appropriate aliquot of CLEAs that guaranteed a linear response. To do so the fraction of CLEAs assayed was 11.4 ± 0.4 mg (Column “Mass of CLEA assayed (aliquot)” in Table 3) whose PP content would fall in the linear range (mass of PP ~ 3 mg) even if *all* PP used in the synthesis protocol had got trapped in the mass of CLEAs recovered. Aliquots for assaying the activity of the supernatant and washings were chosen making similar assumptions. The mass of PP present in supernatant and washings aliquots was then obtained from activity-mass of PP graphs, and total PP lost in supernatants and washings could be inferred (0.57 ± 0.28 mg PP) (Column “Total mass of PP lost in supernatant and washings” in Table 3). Subtraction of this last value from the initial mass of PP used, led to the total mass of PP present in CLEAs (23.2 ± 0.28 mg); which implies that only 24% wt/wt of the mass of the CLEAs synthesized belongs to PP. On the other hand, comparison of the mass of PP recovered in CLEAs with the total mass of PP used in the synthesis protocol evidences that 96 ± 1% of the protein used in the synthesis got trapped in the aggregates. To determine the specific activity of CLEAs the mass of PP present in the aliquot of CLEAs assayed needed to be inferred (Column “Mass of PP in aliquot of CLEA assayed” in Table 3). Since the content of PP is assumed to be constant all throughout the mass of CLEAs recovered, the mass of PP present in the aliquot of CLEAs assayed could be simply obtained once the ratio of the mass of CLEAs assayed to the total mass of CLEAs recovered was known (Columns “Mass of CLEA assayed (aliquot)”/“Total mass of CLEAs recovered” in Table 3). Finally, the specific activity of CLEAs (7.0 ± 0.2 μmol/min-mg) (Column “Specific activity of CLEA” in Table 3) was determined by dividing the registered activity of CLEAs aliquot (19.5 ± 1.15 μmol/min) (Column “Activity of CLEA” in Table 3) by its proportional content of PP (2.8 ± 0.1 mg) (Column “Mass of PP in aliquot of CLEA assayed” in Table 3). The specific activity of native enzyme (62.9 μmol/min-mg) was calculated from the slope of the linear zone of activity-mass of PP (Figure 2, TLL data). The ratio of specific activities allowed the proper calculation of the recovered activity of CLEAs (11.1%) (Column “Recovered activity of CLEA” in Table 3), by comparison with free enzyme under *exactly the same conditions* (including the mass of PP assayed in both cases).

## Discussion

### Determination of precipitable protein (PP) in commercial enzyme solutions

Determination of the specific activity of free enzymes for proper comparison with their cross-linked counterparts, requires the knowledge of the protein content of commercial enzyme preparations. At this point, and due to the complexity of commercial solutions, it is worth stating the difference between commercial preparation, enzyme, protein, lipase and biocatalyst. The term enzyme is often used to refer to the commercial preparation supplied by the provider, even if commercial preparations contain other components different from enzymes such as sugars, nucleic acids, preservatives, etc. In reference to lipases, they are a family of enzymes and as such they are proteins. However, not all proteins present in commercial enzyme preparations are lipases, since they may contain other classes of enzymes (i.e. esterases) which contribute to protein content. In commercial preparations there may also be non-enzymatic proteins. The previous is evidenced in the work of Hernáiz et al. (1994) in which for a commercial preparation of *Candida rugosa* lipase authors determined a protein content of 22%, from which only 3% corresponded to lipase. In the present manuscript precipitable protein, PP, refers to the total mass value obtained by the optimized precipitation method applied to the commercial enzyme preparation, even when it may include other compounds. Lipase is part of the total protein amount and is considered to be the responsible for the catalytic activity registered in triolein hydrolysis. In reference to “biocatalysts”, in this contribution all preparations with enzymatic activity will be mentioned as biocatalysts. Then, the commercial lipase or enzyme solutions or powders are biocatalysts, and CLEAs are also biocatalysts. Immobilized biocatalysts are considered separately from soluble/non immobilized biocatalysts. To sum up, the following sequence from higher to lower complexity in composition in a soluble biocatalyst may be considered: soluble biocatalyst=commercial liquid enzyme preparation → precipitable protein (PP) content → enzyme content → lipase content. To avoid confusions between enzyme, protein and lipase, precipitable protein was selected as the mass basis to calculate the specific activities. The higher the amount of PP is in the reaction media, the higher the amount of lipase.

Even if there are many reports of the use of Lowry, Biuret, Bradford or bicinchoninic methods for the determination of protein content, the precipitation method used in this contribution was the chosen one since it is an adaptation of the CLEAs´ synthesis protocol (aggregation and precipitation, but with no addition of cross-linker). Besides, a recent work from some of us demonstrated that Bradford/Lowry/Biuret are not adequate because albumin -the protein generally used for calibration of those methods-, shows a different response to buffer concentration and different aggregation-concentration behavior than the proteins to be quantified (lipases) (Lassalle et al. 2011). Since the aggregation status of each protein solution affects its answer in the UV/Visible spectra, the use of albumin as calibration protein is arguable. Even more, recent protocols on the topic also warn against the impact of the protein solution composition (buffer, ionic force, etc.) on the results of these traditional methods of protein quantification (Olson and Markwell 2007). The previous observations about the questionable validity of Bradford, Lowry and Biuret methods may justify the importance differences found in the activity of TLL in triolein hydrolysis registered in the current contribution (62.9 μmol/min-mg_PP_)), and the one reported by Rodriguez et al. (2010) (3900 μmol/min-mg), who used Bradford with BSA as standard for protein determination. Due to the different aggregation behavior of BSA and the target lipase in aqueous solutions including buffers, Bradford method may give protein values lower than the real, and thus lead to specific activity values higher than real. Considering the lack of information on enzymatic commercial solutions composition, proper quantification of PP requires a general procedure that does not depend on the solution composition and their components concentrations (buffers, sugars, carbohydrates, inorganic salts, other unknown components including other proteins and other enzymes).

### Determination of linear ranges in activity – mass of PP curves

In enzyme-mediated reactions the increase of biocatalyst concentration does not indefinitely lead to a continuous increment of activity; but instead, at sufficiently high biocatalyst loads, nearly constant values are attained. The previous is clearly shown in the Ph. D. Thesis of Arroyo (1995) devoted to the study of the hydrolysis of triolein and tributyrin catalyzed by lipases from *Candida rugosa* and *Candida antarctica B*. In the case of Arroyo (1995) the maximum concentration of lipase for which a linear activity response was verified was 0.4 mg/mL for CRL in the hydrolysis of triolein (Buffer Tris/HCl 0.001 M – pH = 7.5, T = 35°C, substrate concentration = 2.275 mg triolein/mL, stirring rate= 500 rpm, reaction time = 10 min, interval of CRL concentrations assayed = 0 – 1.25 mg/mL), and 0.15 mg SP525/mL for CALB in the hydrolysis of triacetin (Buffer Tris/HCl 0.001 M – pH = 7.0, T = 37°C, substrate concentration = 11.6 mg triacetin/mL, stirring rate = 500 rpm, reaction time = 10 min, interval of SP525 concentration assayed = 0 - 0.275 mg/mL). In the mentioned reference the author used activity versus enzyme concentration data for selecting an appropriate amount of the immobilized enzyme in the determination of its retained activity with respect to native enzyme. Unfortunately, this kind of analysis is very rare in the published literature. It is also worth noting that similar graphics using Units or volume of liquid enzyme preparation in the x-axis are not useful. Due to the complexity of commercial solutions of enzymes, it is possible that equal masses of lipase/protein are found in different Units or volumes of the commercial solutions. Moreover, Units are dependent on the test reaction and conditions selected by the supplier, which are not necessarily the same for all lipases compared. In fact, Units informed by the supplier may have even been obtained in a type of reaction different from the one of interest (for example interesterifications or esterifications instead of a hydrolysis reaction as the one used as test herein- see Table 1 for examples). It then follows that comparisons of lipases in terms of Units is definitely not recommended since it would probably lead to wrong conclusions about their relative performance in a target reaction.

A review of specific literature evidences that catalytic activity of CLEAs of lipases has been determined by use of a wide variety of reactions, mainly esterifications and hydrolysis. Yu et al. (2006) used the esterification of lauric acid with 1-propanol in isooctane to test the activity of CLEAs of *Candida rugosa* lipase. Shah et al. (2006) monitored the hydrolysis of *p-*nitrophenyl palmitate (PNP) catalyzed by CLEAs of lipase from *Pseudomonas cepacia* through changes of absorbance at 410 nm. Hara et al. (2008) assayed the enzymatic activity of CLEAs of *Pseudomonas cepacia* lipase by hydrolysis of *p-*nitrophenyl acetate (PNA) as a substrate in aqueous solution. Irrespectively of the reaction test used to monitor CLEAs activity, a linear relationship between activity and protein concentration should be verified prior to any other assay. It is worth noting that activity could be similarly plotted versus PP concentration, just by dividing the mass of PP by the total volume of reaction. Considering that the volume introduced by the commercial enzyme aliquot is generally much lower than the total volume of reaction, the target figure would look practically identical. Otherwise, the effect of dilution should be considered. In the current contribution, activity-PP mass/concentration curves included in Figure 2 were of great help for the determination of the appropriate quantities of CLEAs, supernatant aliquots, and washings aliquots to be assayed that guaranteed a linear responses. Moreover, these data allowed inferring the PP content of supernatant and washing aliquots, and thus determine the total amount of PP lost. Collection of these data is also especially important in the case of contributions which quantify enzyme immobilization by following the reduction of enzymatic activity in the supernatant of the immobilization solution (Schoevaart et al. 2004; Kumar et al. 2010). That type of assay requires that the activity measurements are performed using supernatant dilutions that fall in the linear activity-mass of PP zones; otherwise, decreased protein content assigned to immobilization could definitely not be correlated with activity reduction in supernatants. Moreover, since linear activity-biocatalyst concentration ranges depend on the particular enzyme assayed, individual determination of linear intervals is required prior to the calculation of specific activity.

The existence of the “plateaus” shown in Figure 2 may be justified by several reasons. To begin with, products of reaction such as alcohols and oleic acid have been proposed to act as inhibitors (Gaur et al. 2006). At sufficiently high catalyst loads, high production of fatty acids and glycerol resulting from hydrolysis could lead to lipase inhibition and partial/total reduction of enzymatic activity. Another possible explanation for plateaus may be the agglomeration of enzymatic proteins at high loadings. Intrinsic tryptophan fluorescence, surface hydrophobicity and dynamic light scattering (DLS) have been used to demonstrate that lipase aggregation in aqueous media dramatically increases with enhanced protein concentration (Liou et al. 1998). Aggregation of lipases in aqueous media (Liou et al. 1998; Kim and Lee 1998), and also in non-aqueous environments where attractive forces between enzymes are stronger than the ones responsible for their dispersion, has been reported (Bloomer et al. 1992). Formation of aggregates reduces the real concentration of enzyme available for contacting substrates. The enzymes molecules on the outer surface of aggregates are exposed to high substrate concentrations, but mass transport into a particle of clumped catalysts can severely limit the concentration of substrates inside the particles (Foresti et al. 2005). Then, just the fraction of the catalyst that remains on the outer surface of the agglomerates is truly available for catalysis, being the measured specific activity seriously reduced. Plateau-type patterns found in this contribution have also been reported for the same commercial preparations of CRL, PFL and CALB (native, immobilized on polypropylene and/on chitosan supports) in the solvent-free esterification of oleic acid and ethanol (Foresti and Ferreira 2007; Foresti et al. 2005). In the referenced articles, linear relationships between catalyst mass and activity were found to cover a short range of catalyst concentrations.

Besides, the appearance of plateaus shown in Figure 2 could be attributed to “interface quality”, a concept thoroughly discussed in the recent work of Reis et al. (2010). Lipases, like many other proteins, are interfacially active. However, hydrolysis of triglycerides generates highly interfacial active molecules that may compete with the enzyme for the interface and/or modify the protein via molecular interactions. Both potential mechanisms can have an impact on lipase catalysis and may play a key role on the catalytic activity registered. Reis et al. (2010) demonstrated with experimental work and theoretical modeling that monoglycerides give considerably lower values of interfacial tension than lipases, which implies that monoglycerides can expel a lipase from the interface even at relatively low concentration and in the presence of the other hydrolysis products. In this context, it could be hypothesized that at sufficiently high enzyme load the hydrolysis level could be such that interaction between enzyme and substrate is not possible, and thus hydrolytic activity is not increased with protein loading in the expected fashion.

### Determination of precipitable protein (PP) in CLEAs by mass balance, and calculation of their recovered activity

Possible methods to determine the protein content of CLEAs could be the well-known Lowry, Biuret or Bradford assays, although some limitations of these methods have been previously discussed for free enzymes. However, for the quantification of protein present in CLEAs an additional difficulty appears: Lowry, Biuret or Bradford assays would only quantify the fraction of protein that is available for reacting with the colorimetric reagents used in those methods. The previous means that protein present in the core of the aggregates, or in any zone of difficult access, would not be quantified by the mentioned assays and thus specific activity will rise. An alternative option for quantifying total protein is to calculate it from a mass balance that includes the initial amount of protein used for CLEAs synthesis as well as the total protein that remained in the supernatant and washing steps performed after CLEAs isolation.

Results of the protocol hereby presented were included in Table 3 and further detailed for the synthesis of CLEAs of TLL synthesized with 50 mg of cross-linker (see Results section). Results showed that when 50 mg of glutaraldehyde were used in the cross-linking step, comparison of CLEAs specific activity (7.0 ± 0.2 μmol/min-mg) with the specific activity of free lipase (62.9 μmol/min-mg) gave a recovered activity of CLEAs of 11.1%. It is interesting to note that if linear ranges had not been determined, and thus higher amounts of free enzyme than 3 mg had been used (i.e. 20 mg), the specific activity of native enzyme would have fallen to 9.4 μmol/min-mg. As a consequence, it would have been concluded that the synthesized CLEAs had retained nearly 75% of the specific activity of the free enzyme (instead of 11.1%). If even higher amounts of free lipase had been used (i.e. 50 mg), the relative recovered activity of CLEAs would have been 185% and it would have been concluded that the CLEAs synthesis protocol was a complete success. The previous are just two very simple examples of the importance of an appropriate calculation of the recovered activity of CLEAs, since extremely erroneous conclusions can be easily drawn. Evidently, quantification of protein content in commercial lipase solutions and proper determination of linear activity-mass of protein ranges are key points for calculating the fraction of specific activity retained by CLEAs, and as such they need to be taken into account in the early stages of biocatalyst development.

## Competing interests

The authors declare that they have no competing interests.
